# Acute and chronic histopathological findings in renal biopsies in COVID-19

**DOI:** 10.1007/s10238-022-00941-x

**Published:** 2022-11-18

**Authors:** Meint Volbeda, Daniela Jou-Valencia, Marius C. van den Heuvel, Jan G. Zijlstra, Casper F. M. Franssen, Peter H. J. van der Voort, Jill Moser, Matijs van Meurs

**Affiliations:** 1 Department of Critical Care, University of Groningen, University Medical Center Groningen, Hanzeplein 1, 9713 GZ Groningen, The Netherlands; 2 Department of Pathology and Medical Biology, Pathology Section, University of Groningen, University Medical Center Groningen, Groningen, The Netherlands; 3 Department of Nephrology, University of Groningen, University Medical Center Groningen, Groningen, The Netherlands; 4 Department of Pathology and Medical Biology, Medical Biology Section, Laboratory for Endothelial Biomedicine and Vascular Drug Targeting Research, University of Groningen, University Medical Center Groningen, Groningen, The Netherlands

**Keywords:** Acute kidney injury, COVID-19, Histopathology, Postmortem, Renal biopsy

## Abstract

The dominant ICU admission diagnosis of COVID-19 patients is respiratory insufficiency, but 32–57% of hospitalized COVID-19 patients develop acute kidney injury (COVID-AKI). The renal histopathological changes accompanying COVID-AKI are not yet fully described. To obtain a detailed insight into renal histopathological features of COVID-19, we conducted a review including all studies reporting histopathological findings of diagnostic and postmortem kidney biopsies from patients with COVID-19 published between January 1, 2020, and January 31, 2021. A total of 89 diagnostic and 194 postmortem renal biopsies from individual patients in 39 published studies were investigated and were included in the analysis. In the diagnostic biopsy group, mean age was 56 years and AKI incidence was 96%. In the postmortem biopsy group, mean age was 69 years and AKI incidence was 80%. In the diagnostic biopsy group, the prevalence of acute glomerular diseases was 74%. The most common glomerular lesions were collapsing focal segmental glomerulosclerosis (c-FSGS) in 54% and thrombotic microangiopathy (TMA) in 9% of patients. TMA was also found in 10% of patients in the postmortem biopsy group. The most common acute tubular lesions was acute tubular necrosis (ATN) which was present in 87% of patients in the diagnostic and in 77% of patients in the postmortem biopsy group. Additionally, we observed a high prevalence of preexisting chronic lesions in both groups such as atherosclerosis and glomerulosclerosis. Histopathological changes in renal biopsies of COVID-19 patients show a heterogeneous picture with acute glomerular lesions, predominantly c-FSGS and TMA, and acute tubular lesions, predominantly ATN. In many patients, these lesions were present on a background of chronic renal injury.

**Graphical abstract:**

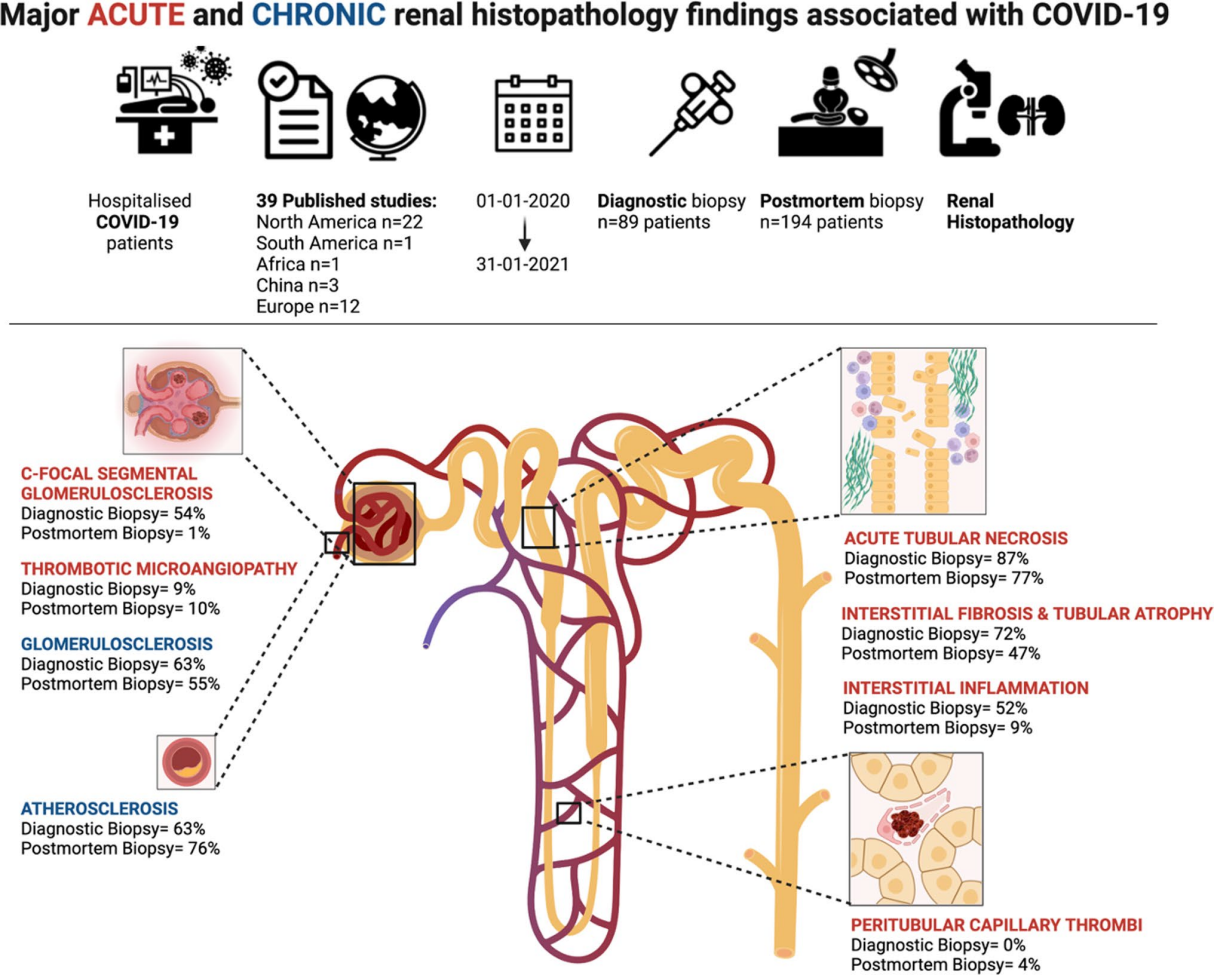

**Supplementary Information:**

The online version contains supplementary material available at 10.1007/s10238-022-00941-x.

## Introduction

The COVID-19 pandemic has resulted in an overwhelming number of hospital and ICU admissions worldwide. While the admission diagnosis is most often respiratory insufficiency, 32–57% [1–4] of patients are hospitalized because of COVID-19 develop acute kidney injury (COVID-AKI). Patients with COVID-AKI have an increased mortality risk compared to COVID-19 patients without AKI (52 vs. 26%)[5].

The mechanisms that lead to the development of COVID-AKI are not yet fully understood [6]. We and others have investigated small case series of renal biopsies in which histological and gene expression profiles in COVID-AKI are reported [7, 8]. In our small case series, COVID-AKI was associated with extensive acute tubular necrosis (ATN), peritubular thrombi, distinct endothelial responses and different renal injury biomarker levels compared to sepsis AKI [7].

In this review, we summarized the renal histopathological features of 283 individual adult COVID-19 patients using data extracted from 39 peer-reviewed published papers. We report differences and similarities between diagnostic biopsies and postmortem findings and discuss the implications of these findings regarding our understanding of COVID-AKI.

## Methods

### Eligibility criteria

Articles published between January 1, 2020, and January 31, 2021, reporting microscopic findings of diagnostic or postmortem kidney biopsies in adult human patients with confirmed SARS-COV-2 infection during the first three COVID-19 waves were reviewed. This inclusion period was chosen since after this period treatments were introduced in the clinic such as corticosteroids [9], antiviral medication and monoclonal antibodies that may affect the natural course of COVID-AKI and thus the biopsy findings. Publications in languages other than English were excluded. No restrictions were applied with respect to study design. Biopsies form kidney transplant recipients were excluded.

### Search strategy

We searched PubMed/Medline and Google scholar. The search items are listed in Table S1. We also hand-searched the reference lists of the results of the electronic search for additional studies.

### Study selection

**Fig. 1:**
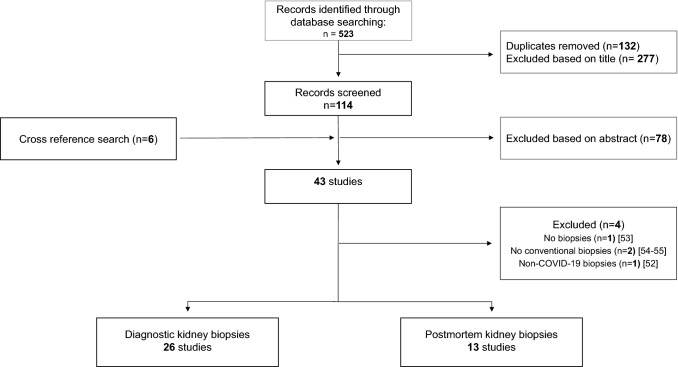
Flow diagram study selection

Titles and abstracts were screened for eligibility based on inclusion/exclusion criteria by one author (DJV). Articles which were deemed suitable were subsequently screened by MV, DJV, MvM and JM. Studies were only included if all these authors agreed and, next, categorized according to type of kidney biopsy: diagnostic or postmortem (Fig. 1).

### Data collection

Characteristics of individual patients in case series and case reports were extracted independently by two authors (DJV and MV). In case the reported data were incomplete, the corresponding authors of the publication were contacted by email with the request to provide additional information. When additional data were received from authors, these data were screened and included if it contributed to the analysis. A summary of patient characteristics is shown in Table 1. A detailed overview of the diagnostic biopsy and postmortem biopsy studies are shown in Table S2 and S3, respectively.

**Table 1:** Baseline characteristics

	Patients with diagnostic kidney biopsy *n* = 89	Not reported	Patients with postmortem kidney biopsy *n* = 194	Not reported	P-value
Treated on critical care (*n*)	36% (15/42)	47	72% (68/94)	100	< 0.001
Age (years) (mean ± SD)	56 (12.8)	0	69 (10.7)	0	< 0.001
Female % (*n*)	31% (28)	0	32% (63)	0	NS
Race % (*n*)		1		123	
Black	68% (60/88)		13% (9/71)		N.a.p.^2^
Hispanics	10% (9/88)		46% (33/71)		
Asian	6% (5/88)		0% (0/71)		
White	16% (14/88)		38% (27/71)		
Other	0% (0/88)		3% (2/71)^1^		
Diabetes mellitus (*n*)	31% (26/83)	6	32% (59/187)	7	NS
Cardiac disease (*n*)	17% (14/83)	6	28% (53/187)	7	NS
Hypertension (*n*)	70% (58/83)	6	63% (105/166)	28	N.a.p.^2^
Chronic kidney disease (*n*)	13% (11/83)	6	18% (27/153)	41	N.a.p.^2^
COPD (*n*)	2% (2/83)	6	14% (20/146)	48	N.a.p.^2^
Obesity (*n*)	25% (21/83)	6	21% (31/149)	45	N.a.p.^2^
Vascular disease (*n*)	5% (4/83)	6	8% (9/117)	77	N.a.p.^2^
Dyslipidemia (*n*)	14% (12/83)	6	11% (13/117)	77	N.a.p.^2^

*N.a.p.*  no analysis performed. ^1^2 Patients in the study by Santoriello [16] self-identified their race as ‘other’. ^2^no statistical analysis performed because of the high proportion of not reported data in the postmortem biopsy group

### Data analysis and statistics

Statistical analysis was performed using IBM SPSS Statistics for Windows, version 26.0 (Armonk, NY, USA).

Patients with a kidney transplant or those without renal biopsy data were excluded from analysis. Renal biopsy findings in individual patients were analyzed and categorized in chronic and acute lesions, specific diagnoses and the localization of the lesions by DJV and MV based on the description in the publications and/or the additional information provided by the corresponding authors. To avoid misinterpretation, the renal pathologist, MvdH, was consulted to verify the analyses and the categorization of renal biopsy findings, if available via microscopic images provided in the publications. Differences between diagnostic and postmortem kidney biopsy groups were analyzed by Chi-square test and Mann–Whitney U test. *p* values < 0.05 were considered significant.

The presence of hematuria was defined as > 3 erythrocytes per high power field (HPF) or > 14 erythrocytes per µl according to the 2010 guideline hematuria of the Dutch Association of Urology [10]. If the number of urinary erythrocytes in an individual patient was not mentioned, the presence or absence of hematuria was based on the definition of hematuria used in the case series or case report in which the patient was described. Proteinuria was defined and categorized according to the Kidney Disease Improving Global Outcome (KDIGO) clinical practice guideline for the evaluation and management of chronic kidney disease [11]. Nephrotic range proteinuria was defined as proteinuria > 3.5 g/day (or urinary protein-to-creatinine ratio > 2000 mg/gram or 200 mg/mmol) according to the KDIGO Guideline [11]. AKI and AKI stages were defined according to the KDIGO clinical practice guideline for AKI [12] and were derived from serum creatinine levels and/or urine output and/or need for renal replacement therapy (RRT). ATN was scored as present or absent.

In case of a possible discrepancy between the published data and additional data received from the author, published data were used.

Several studies only reported the proportion of patients with specific renal lesions without showing the individual patient data [13–17]. To be able to include these patients in our analysis, we created a group of ‘individual patient records’ in which the published frequencies of findings were attributed to a corresponding amount of ‘individual patient records.’ For example, when in a case series of 10 patients without individual patient data 30% of patients had diabetes mellitus (DM) and 70% of patients had atherosclerosis, 10 ‘individual patient records’ were created and DM was reported positive in the first 3 ‘individual patient records’ and atherosclerosis in the first 7 ‘individual patient records.’ Naturally, these data can only be used for descriptive purposes. When for a group of patients only a mean value was given for a specific variable, this mean value was used for all ‘individual patient records.’ When no mean value was available, the median value was used (when available).

## Results

The literature search yielded a total of 523 articles from which 132 were duplicates and 277 articles were excluded based on the title alone (Fig. 1). Subsequently, 78 articles were excluded based on the abstract and 6 were additionally included based on cross-reference search resulting in 43 selected studies [13–55]. Four studies were excluded because they lacked a description of histopathologic biopsy findings or focused on non-COVID-19 renal biopsies [52–55], resulting in 26 studies [18–43] on diagnostic and 13 studies [13–17, 44–51] on postmortem renal biopsies. These articles described biopsy results of 1 to 42 patients (Table S2 and S3). Studies were from various countries but most originated from North America, China and Europe. All diagnostic biopsy studies reported individual patient data (Table S2), whereas 5 postmortem studies comprising a total of 102 patients did not report individual patient data (Table S3) [13–17]. Eight patients in the diagnostic study group underwent biopsies of a kidney transplant and were excluded, and one patient was described in two studies [33, 40] and was therefore only included once in our analysis [33] (Table S2). Similarly, 1 patient in the postmortem group was excluded due to kidney transplant [44]. Eight other patients were excluded because kidney biopsy results of these patients were not described in the publication (Table S2) [13, 45, 49]. In this review therefore, 89 diagnostic and 194 postmortem kidney biopsy patients were included.

### Baseline characteristics

Patients in the diagnostic biopsy group were on average 56 years old compared to 69 years in the postmortem biopsy group (*p* < 0.001) and were less likely to need ICU treatment (36 versus 72%, (*p* < 0.001)) (Table 1). Thirty-one percent of patients in the diagnostic biopsy group were female compared to 32% in the postmortem biopsy group. In the diagnostic biopsy group, 68% of patients were black. Unfortunately, race was not reported in 63% of patients in the postmortem biopsy group. The incidence of diabetes mellitus was 31% in diagnostic biopsy group and 32% in the postmortem biopsy group (ns). In the diagnostic biopsy group, the incidence of pre-existing cardiac disease was 17% compared to 28% in the postmortem biopsy group (ns) (Table 1). The incidences of preexisting chronic diseases in the diagnostic biopsy group were 70% for hypertension, 25% for obesity, 14% for dyslipidemia, 5% for vascular disease and 2% for COPD. It is of note that 13% of patients in the diagnostic biopsy group had a history of chronic kidney disease. Additional incidences of chronic diseases in the postmortem biopsy group could not be reliable estimated since a high proportion of patients had missing values ranging from 14% for hypertension to 40% for vascular disease and dyslipidemia (Table 1).

### Diagnostic biopsy group

#### Clinical manifestations

In the diagnostic biopsy group, 96% of patients had AKI according to the KDIGO criteria; 83% of patients had AKI stage 3 and 68% of patients received RRT (Table 2). In the diagnostic biopsy group, 93% of patients had KDIGO stage A3 proteinuria, 73% of patients had nephrotic range proteinuria, and 48% had hematuria (Table 2).

**Table 2:** Clinical AKI manifestations

	Patients with diagnostic kidney biopsy (*n* = 89)		Patients with postmortem kidney biopsy (*n* = 194)	
		Not reported		Not reported
KDIGO AKI (*n*)	96% (85/89)	0	80% (87/109)	85
Stage 1	4% (4/89)		16% (18/109)	
Stage 2	8% (7/89)		16% (17/109)	
Stage 3	83% (74/89)		48% (52/109)	
RRT (*n*)	68% (59/87)	2^1^	28% (38/135)	59
Proteinuria (according to KDIGO^2^) (*n*)		0		138
A1	0% (0/89)		4% (2/56)	
A2	1% (1/89)		2% (1/56)	
A3	93% (83/89)		63% (35/56)	
Not quantifiable	6% (5/89)		32% (18/56)	
Nephrotic range proteinuria (according to KDIGO^3^) (*n*)	73% (65/89)	0	3% (1/36)	158
Not quantifiable	7% (6/89)		33% (12/36)	
Hematuria (*n*)	48% (40/84)	5	62% (34/55)	139
Not quantifiable	4% (3/84)			

No comparisons were performed between the diagnostic and postmortem biopsy group because of the high proportion of not reported data in the postmortem biopsy group. ^1^In the study of Akilesh [18] the authors state that it is unknown whether patient 2 and 4 received RRT.^2^KDIGO clinical practice guideline for the evaluation and management of chronic kidney disease [11]. ^3^Apart from missing data, the incidence of nephrotic range proteinuria might be underestimated, since proteinuria could not be quantified in 6 patients in the diagnostic kidney biopsy group and 12 patients in the postmortem biopsy group. KDIGO = Kidney Disease Improving Global Outcome, AKI = Acute Kidney Injury, RRT = Renal Replacement Therapy

#### Renal biopsy results

**Fig. 2:**
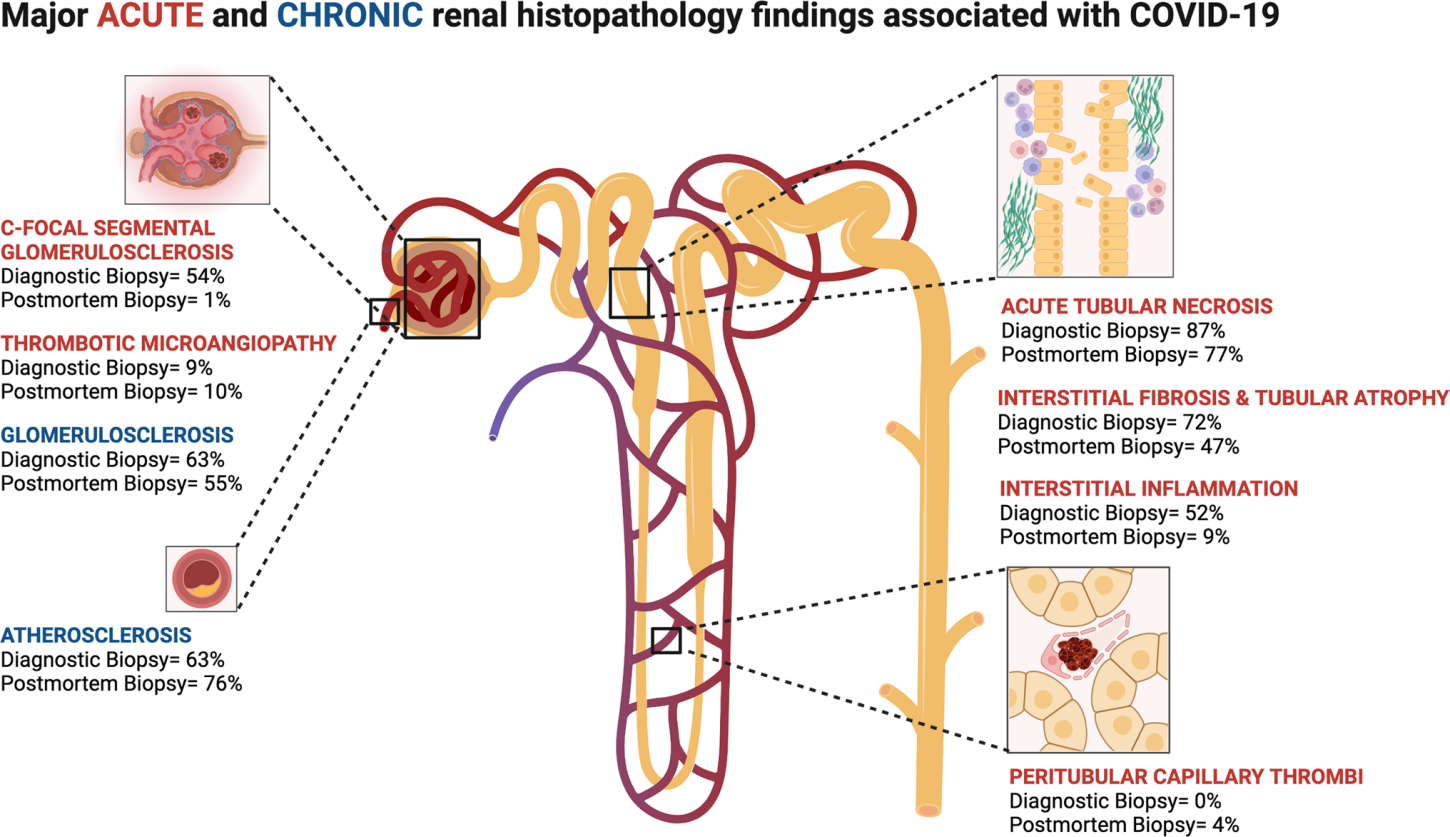
Major acute and chronic renal histopathology findings associated with COVID-19

Acute glomerular disease was found in one or more forms in 74% of patients. The most prevalent lesions were collapsing focal segmental glomerulosclerosis (c-FSGS) in 54% of patients and thrombotic microangiopathy (TMA) in 9% of patients (Table 3). Acute tubulo-interstitial disease was found in one or more forms in 94%, with a prevalence of 87% of acute tubular necrosis (ATN), 72% of interstitial fibrosis tubular atrophy (IFTA), 52% of interstitial inflammation, 2% of pigment casts and 1% oxalate nephropathy (Table 3). Acute vascular disease was not found besides glomerular microthrombi which were attributed to TMA. Chronic lesions were found in 83%, with a prevalence of 63% of glomerulosclerosis and 63% of atherosclerosis (Table 3). A summary of the biopsy findings is also shown in Fig. 2.

**Table 3:** Biopsy results

	Patients with diagnostic kidney biopsy (*n* = 89)	Patients with postmortem kidney biopsy (*n* = 194)	*p*-value
Time to postmortem biopsy in hours (overall range)	N.a	1–186	N.a
(Semi) acute lesions % (n)			
*Glomerular*	*74% (66)*	*10% (20)*	< *0.001*
Collapsing FSGS	54% (48)	1% (1)	< 0.001
Non-collapsing FSGS	2% (2)	0% (0)	0.036
Thrombotic microangiopathy	9% (8)	10% (20)	NS
IgA nephropathy	3% (3)	1% (1)	NS
Membranous nephropathy	3% (3)	0% (0)	0.010
Minimal change disease	2% (2)	0% (0)	0.036
Crescentic glomerulonephritis	2% (2)	0% (0)	0.036
Lupus nephritis class IV/V	1% (1)	0% (0)	NS
Anti-GBM nephritis	1% (1)	0% (0)	NS
Post-infectious nephropathy	1% (1)	0% (0)	NS
*Vascular*			
Microtrombi	8% (7)	12% (23)	NS
Glomerular	8% (7)	9% (17)	NS
Peritubular	0% (0)	4% (7)	NS
*Tubulo-interstitial*	*94% (83)*	*93% (181)*	*NS*
ATN	87% (77)	77% (131)^1^	< 0.001
TIN	4% (4)	1% (1)	0.018
IFTA	72% (64)	47% (92)	< 0.001
Interstitial inflammation	52% (46)	9% (17)	< 0.001
Pigment casts	2% (2)	8% (16)	NS
Cast nephropathy	1% (1)	0% (0)	NS
Oxalate nephropathy	1% (1)	2% (3)	NS
*Mesangial expansion*	*12% (11)*	*5% (10)*	*0.032*
Chronic lesions % (n)	83% (74)	86% (167)	NS
*Glomerular*			
Glomerulosclerosis	63% (56)	55% (107)	NS
Atherosclerosis	63% (56)	76% (148)	0.020
Diabetic nephropathy	11% (10)	7% (14)	NS
FSGS	2% (2)	2% (3)	NS
IgA nephropathy	1% (1)	1% (1)	NS
Membranous nephropathy	1% (1)	0% (0)	NS
Nephrocalcinosis	0% (0)	2% (4)	NS

Incidences are based on the description of biopsy results in the original articles. Possibly specific biopsy features were not reported in the original articles, which could have led to underestimation of incidences. ^1^ATN could not be assessed in 23 biopsies due to autolysis

*n.a.*, not applicable, *FSGS* = focal segmental glomerulosclerosis, *anti-GBM disease*, anti-glomerular basement membrane disease, *ATN*, acute tubular necrosis, *TIN*, tubulo-interstitial nephritis, *IFTA*, interstitial fibrosis tubular atrophia

#### Correlation between clinical manifestations and renal biopsy results

In the diagnostic biopsy group, patients with c-FSGS had a mean age of 55 years, were male in 75% and black in 94% of cases and had a prevalence of KDIGO AKI stage 3 of 92%, KDIGO A3 proteinuria of 100%, nephrotic range proteinuria of 85% and required RRT in 72%. Patients with TMA had a mean age of 58 years, and all had KDIGO AKI stage 3 (100%) combined with RRT requirement (100%). DM nephropathy was found in 35% of patients with DM. No significant association was found between glomerulosclerosis, atherosclerosis or diabetic nephropathy and the occurrence of ATN (data not shown).

### Postmortem biopsy group

#### Clinical manifestations

In the postmortem biopsy group, data on clinical manifestations were frequently missing. In this group, 80% of patients (87 of 109 patients with sufficient data) had AKI, 48% of patients (52 of 109 with sufficient data) had AKI stage 3, and 28% of patients (38 of 135 with sufficient data) received RRT (Table 2). The prevalence of KDIGO A3 proteinuria was 63% (35 of 56 with sufficient data), 1 patient had nephrotic range proteinuria, and 62% of patients (34 of 55 with sufficient data) had hematuria (Table 2).

#### Renal biopsy results

In the postmortem biopsy group, the interval between death and postmortem kidney biopsy varied between 1 and 186 h (Table 3 and Table S3). Acute glomerular disease was found in 10% of patients with a prevalence of 1% of c-FSGS and 10% of TMA (Table 3). Acute tubulo-interstitial disease was found in 93%, with a prevalence of 77% ATN, 47% IFTA, 9% of interstitial inflammation, 8% of pigment casts and 2% of oxalate nephropathy (Table 3). Acute vascular disease was found as peritubular (4%) and glomerular (9%) microthrombi. Glomerular thrombi were attributed to TMA. Chronic lesions were found in one or more forms in 86%, with a prevalence of 55% of glomerulosclerosis and 76% of atherosclerosis (Table 3). A summary of the biopsy findings is also shown in Fig. 2.

#### Correlation between clinical manifestations and renal biopsy results

In the postmortem biopsy group, the analysis of the association between clinical manifestations and renal biopsy data was complicated by missing clinical information ranging from 30% for RRT to 81% for nephrotic range proteinuria and by the absence of individual patient data in 52% of patients [13–17]. No further analysis on c-FSGS was performed since only one patient without individual patient data had c-FSGS. Patients with TMA (6 of 20 with individual patient data) had a mean age of 78 years and prevalence of both AKI stage 3 and need for RRT in 17% of patients (1/6 (AKI stage and RRT need was unknown in 1 patient).

**Fig. 3:**
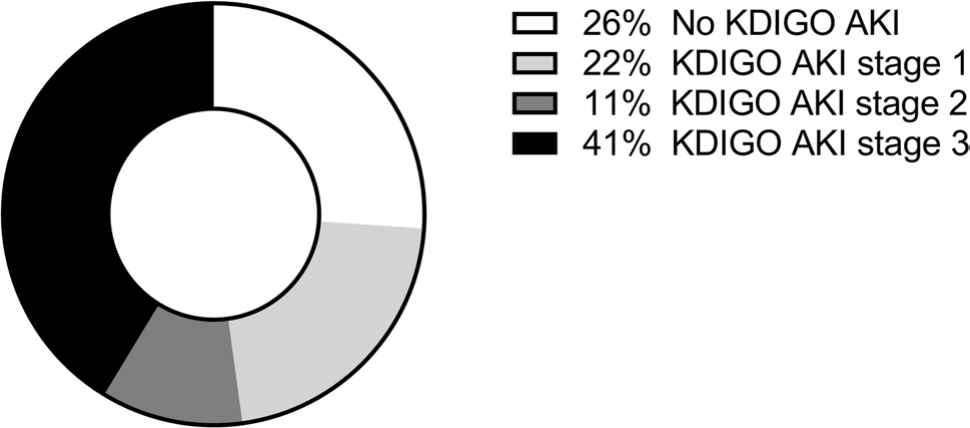
Prevalence of KDIGO AKI stages in patients in the postmortem biopsy group with ATN on renal biopsy

The extent of ATN was not associated with the AKI classification in patients with complete individual patient data (46 of 194 patients) (Fig. 3). In 42% (82 of 194) of patients in which the required individual patient data as well as non-autolytic biopsy samples were available, no significant association was found between glomerulosclerosis, atherosclerosis, diabetic nephropathy and the occurrence of ATN (data not shown). Diabetic nephropathy (26%) and atherosclerosis (96%) were evident in DM patients with individual patient data (27 of 59), respectively.

### Comparison of clinical manifestations and renal biopsy results between both biopsy groups

A higher prevalence of AKI, AKI stage 3, proteinuria and nephrotic range proteinuria and need for RRT was found in the diagnostic biopsy group compared to the postmortem biopsy group (Table 2). The prevalence of acute glomerular disease was higher in the diagnostic biopsy group compared to the postmortem biopsy group (Table 3). TMA prevalence was comparable in both groups. The overall prevalence of acute tubule-interstitial diseases was equal in both groups; however, the prevalence of ATN, TIN, IFTA and interstitial inflammation was higher in the diagnostic biopsy group (Table 3).

## Discussion

In this review, we found a high prevalence of c-FSGS in the diagnostic biopsy group and a high prevalence of TMA in both the diagnostic and postmortem biopsy group. We also found a high prevalence of ATN in both the diagnostic and postmortem biopsy group. Additionally, we observed a high prevalence of chronic lesions in both biopsy groups such as atherosclerosis and glomerulosclerosis.

c-FSGS was the most frequent acute glomerular lesion in COVID-19 patients in our study. c-FSGS is associated with the presence of a high-risk apolipoprotein L1 (APOL1) genotype, which is defined as the presence of homozygous G1 (G1/G1), G2 (G2/G2) or compound heterozygous G1/G2 risk alleles and has been reported only on chromosomes from persons from African origin [56]. The prevalence of c-FSGS (54%) in the diagnostic biopsy group was much higher compared to the prevalence of 26% that was recently reported by May et al. containing 240 diagnostic kidney biopsies in COVID-19 patients [57] and is likely due to a publication bias also at least in part because of the high proportion of 68% of patients of African ancestry in the diagnostic biopsy group compared to a proportion 45% in the study of May [57].

TMA was found in approximately 10% in both the diagnostic and the postmortem biopsy group. In a recent review by Tiwari, TMA is proposed to play an important role via the development of microthrombi in micro vessels of the kidney [58].

The high prevalence of ATN in both biopsy groups is in accordance with our recent findings in 6 patients with COVID-19 who all had ATN [7]. In a recent postmortem biopsy cohort from Mexico described by Rivero, the incidence was lower but still considerable at 49% [59]. In the diagnostic biopsy group, ATN was frequently accompanied by acute glomerular disease. In our own recent biopsy study, we suggested that ATN could be a consequence of a diminished peritubular flow caused by microthrombi [7]; however, in this current review the incidence of microthrombi in the diagnostic and the postmortem biopsy group was only 8% and 12%, respectively.

In a subset of patients in the postmortem biopsy group, we observed that 26% of patients with ATN on renal biopsy did not have AKI at all. In the study by Rivero also, no clear correlation between ATN and AKI severity was found [59]. Possibly, the renal functional reserve in these patients is large enough to prevent a rise in serum creatinine as a consequence of loss of functional nephrons [60]. Since ATN could not be uniformly assessed, ATN severity might differ significantly.

IFTA was frequently observed in both the diagnostic and postmortem biopsy group. In a recent large study investigating renal histopathology and kidney function, the presence of IFTA was associated with a diminished eGFR [61]. From observational data described in our study, no mechanistic conclusions can be drawn. Interestingly, in an experimental study in which human-induced pluripotent stemcell-derived kidney organoids, a profibrotic response was observed when infecting these organoids with COVID-19 [62]. The authors of this experimental study suggested that AKI and CKD in COVID-19 patients could be a consequence of fibrosis [62]. In this review in both groups, no significant difference between AKI stage in patients with and without IFTA was found.

In both biopsy groups, chronic lesions were frequently observed, which implies a much higher incidence of chronic kidney disease (CKD) than reported in the medical history of the included patients. In general, CKD patients with COVID-19 have a highly increased risk for worsening of renal function and mortality [63]. More specifically, COVID-19 ICU patients with CKD are also known to have an increased mortality risk compared to non-CKD Covid-19 ICU patients [64].

‘Coronavirus like particles’ by electron microscopy (EM) were reported in various studies which we reviewed [15, 21, 28, 35, 44, 46, 47, 49, 51]. However, these EM findings were subsequently seen as a misinterpretation [65]. We therefore have not included these findings in this review. SARS-CoV2 viral protein and/or viral RNA detection was also performed in several included studies [16–20, 24–26, 28, 29, 33, 36–40, 43–51]. The results of these analyses were recently summarized by Hassler et al. [66], and we therefore did not repeat this investigation. Hassler concluded that despite negative results in multiple studies, there are data demonstrating SARS-CoV-2 tropism in kidneys although without evidence for a direct pathophysiological link with AKI [66].

There are important differences between pre- and postmortem renal biopsy conclusions for several reasons: (1) Different patient characteristics: (a) Patients in the postmortem biopsy group were older. (b) Diagnostic renal biopsies were performed because of acute renal failure during the course of COVID-19, and renal biopsies were performed according to established treatment standards compared to postmortem renal biopsies obtained for research from patients who died from COVID-19. (2) Possible loss of tissue integrity (autolysis) in postmortem renal biopsies because of the long duration until biopsies were performed [67]. (3) Postmortem biopsies were obtained after death at varying time points in the course of COVID-19 infections. (4) Renal biopsies are rarely performed in critically ill AKI patients complicating an uniform structured approach and interpretation of the biopsy material [68]. Many studies for example did not mention the presence or absence of microthrombi, and additional Martius Scarlet Blue (MSB) staining technique is necessary for fibrin visualization and to detecting of these small thrombi. Some studies mention calcium oxalate depositions [17, 31, 48, 50], which was in the study by Malhorta interpreted as a consequence of vitamin C administration as part of supplementary treatment of COVID-19 [31]. However, these crystals are only visualized by microscopy with polarized light and will be missed when only examination of digital images of biopsy material is performed.

In order to understand disease-specific mechanisms leading to AKI in COVID-19 future, postmortem biopsy studies need to address the following items: (1) retrieval of postmortem kidney biopsy most preferably within 60 min after death at the bedside in order to perform molecular biology on biopsy samples. Protein, RNA and DNA analysis require this fast sample handling and careful storage to prevent postmortem degradation effects [67]. For conventional histopathological analysis probably, a longer time-frame can be applied before autolysis occurs. However, in our experience postmortem kidney biopsies can easily be performed within 60 min after death [7, 69], and we therefore advice to use the same maximum time-frame of 60 min. (2) Uniform analyses and reporting of biopsy material. (3) Uniform reporting of patient characteristics for detection of specific AKI subtypes. (4) Postmortem biopsies should be performed more often both in COVID-AKI patients and non-COVID-AKI patients in order to discover both similarities and differences between COVID-AKI and non-COVID-AKI.

The strength of this review is the detailed description of renal pathology in a large group of individual patients from different studies worldwide, as well as illustrating the pathological findings in both diagnostic and postmortem kidney biopsies. However, several limitations should be considered. (1) We did not perform the actual pathological re-analyses of the renal biopsies from the different studies but had to use the information in the publication with a varying description and interpretation of the biopsy findings in the different studies. (2) Due to limited clinical data, especially in the postmortem biopsy group which included also five studies without individual patient data [13–17], we could only investigate a few possible associations between clinical data and biopsy findings. Despite these limitations, this review highlights several different renal histopathological findings in COVID-19, which suggests different pathophysiological mechanisms leading to AKI. However, the above mentioned suggestions have to be taken into account to increase knowledge in disease-specific mechanisms leading to AKI in COVID-19.

## Conclusions

Renal biopsies from COVID-19 patients showed a high prevalence of c-FSGS in the diagnostic biopsy group and a high prevalence of TMA in both the diagnostic and postmortem biopsy group. ATN and chronic lesions, such as atherosclerosis and glomerulosclerosis, also had a high prevalence in both biopsy groups. Our findings suggest that different pathophysiological processes may lead to AKI in COVID-19 patients. Future studies need to address the clinical relevance of these findings.

## Supplementary Information

Below is the link to the electronic supplementary material.

Supplementary file1 (DOCX 25 KB)

## Data Availability

The data underlying this article will be shared upon reasonable request to the corresponding author.
