# Supplementary material for: Acute and chronic histopathological findings in renal biopsies in COVID-19
Source: Clin Exp Med. 2022 Nov 18;23(4):1003–14. doi: 10.1007/s10238-022-00941-x (PMC9672628; doi:10.1007/s10238-022-00941-x)
Supplement: Supplementary file 1 — Supplementary file1 (DOCX 25 KB) [file 10238_2022_941_MOESM1_ESM.docx]

Index of tables:

Table S1 Search strategy.

Table S2 Overview of included studies with diagnostic kidney biopsies in Covid-19 patients.

Table S3 Overview of included studies with post-mortem kidney biopsies in Covid-19 patients.

**Table S1.** Search strategy to identify records for potential inclusion in this review.

| **Database** | **Search Strategy** |
| --- | --- |
| PubMed | (((Acute kidney Injury) OR (acute renal failure)) AND ((COVID 19) OR (SARS-COV-2))) AND (((renal histopathology) OR (kidney histopathology)) OR ((kidney biopsy) OR (renal biopsy))) |
| Google Scholar | ((COVID-19) OR (SARS-COV-2)) AND ((((((renal histopathology) OR (kidney histopathology)) OR (renal pathology)) OR (kidney pathology)) OR (kidney biopsy)) OR (renal biopsy)) |

**Table S2.** Included studies with diagnostic kidney biopsies in Covid-19 patients.

| **First author** | **Country** | **Number of patients** | **Excluded patients in current analysis** | **Individual patient biopsy data** | **Number of included patients with image(s) available for histo-pathologic analysis** |
| --- | --- | --- | --- | --- | --- |
|  |  |  |  |  |  |
| Akilesh [18] | USA | 17 | 3 patients: renal transplant recipients | Yes | 4 |
| Couturier[19] | France | 2 |  | Yes | 2 |
| Dargelos [20] | France | 3 |  | Yes | 2 |
| Deshmukh[21] | USA | 1 |  | Yes | 1 |
| Gaillard [21] | France | 1 |  | Yes | 1 |
| Gupta [23] | USA | 2 |  | Yes | 2 |
| Huang [24] | China | 1 |  | Yes | 1 |
| Kudose [25] | USA | 17 | 3 patients: renal transplant recipients | Yes | 0^1^ |
| Izzedine [26] | France | 2 |  | Yes | 2 |
| Jhaveri [27] | USA | 1 |  | Yes | 1 |
| Kissling [28] | Swiss | 1 |  | Yes | 1 |
| Larsen [29] | USA | 1 |  | Yes | 1 |
| Magoon [30] | USA | 2 |  | Yes | 2 |
| Malhotra [31] | USA | 1 |  | Yes | 1 |
| Malik [32] | USA | 1 |  | Yes | 1 |
| Nasr [33] | USA | 13 |  | Yes | 4 |
| Nlandu [34] | Congo | 1 |  | Yes | 1 |
| Noble [35] | UK | 2 | 1 patient: renal transplant recipient | Yes | 1 |
| Papadimitriou [36] | USA | 2 |  | Yes | 2 |
| Peleg [37] | USA | 1 |  | Yes | 1 |
| Rossi [38] | Italy | 1 |  | Yes | 1 |
| Sharma [39] | USA | 10 |  | Yes | 5 |
| Sharma [40] | USA | 2 | Patient 1 is patient 6 in the study by Nasr [33] | Yes | 2 |
| Shetty [41] | USA | 6 | 1 patient: renal transplant recipient | Yes | 5 |
| Suso [42] | Spain | 1 |  | Yes | 1 |
| Wu [43] | USA | 6 |  | Yes | 2^2^ |

**Legend.** ^1^ selective images are published without references to specific patients, ^2^ additional images are published without reference to specific patients.

**Table S3.** Included studies with post mortem kidney biopsies in Covid-19 patients.

| **First author** | **Country** | **Number of patients** | **Excluded patients and reason for exclusion** | **Individual patient biopsy data** | **Number of included patients with image(s) available for histopathologic analysis** | **Interval to biopsy after death in hours^1^** |
| --- | --- | --- | --- | --- | --- | --- |
| Bradley [44] | USA | 14 | 1 patient: renal transplant recipient. | Yes | 0 | No data |
| Brook [45] | USA | 5 | 2 patients: absence of kidney biopsy | Yes | 1 | 2.5  (1.4-2.8) |
| Diao [46] | China | 6 |  | Yes | 6 | No data |
| Duarte [13] | Brazil | 10 | 2 patients: absence of kidney biopsy | No | 0^2^ | No data |
| Falasca [14] | Italy | 22 |  | No | 0^2^ | No data |
| Farkash [47] | USA | 1 |  | Yes | 0^3^ | No data |
| Golmai [48] | USA | 12 |  | Yes | 0^2^ | 17.5  (1.5-70) |
| Menter [49] | Swiss | 21 | 4 patients: absence of kidney biopsy | Yes | 17 | 24.0  (9-84) |
| Remmelink [50] | Belgium | 16 |  | Yes | 0 | 72-96 |
| Rapkiewicz [15] | USA | 7 |  | No | 2 | No data |
| Santoriello [16] | USA | 42 |  | No | 0^2^ | 21.8  (2.5-186) |
| Schurink [17] | The Nether-lands | 21 |  | No | 0^2^ | 15.0 |
| Su [51] | China | 26 |  | Yes | 0^2^ | 1-6 |

**Legend.** ^1^ median and/or range depending on available data, ^2^ selective images are published without references to specific patients, ^3^ images focus on evidence for direct renal infection with SARS-CoV-2.
